# Rural suicide in Newfoundland and Labrador: A qualitative exploration of health care providers’ perspectives

**DOI:** 10.1371/journal.pone.0306929

**Published:** 2024-08-12

**Authors:** Tyler R. Pritchard, Jennifer L. Buckle, Kristel Thomassin, Stephen P. Lewis

**Affiliations:** 1 Department of Psychology, University of Guelph, Guelph, Ontario, Canada; 2 Department of Psychology, Grenfell Campus, Memorial University of Newfoundland and Labrador, St. John’s, Newfoundland and Labrador, Canada; Fisheries and Oceans Canada, CANADA

## Abstract

**Introduction:**

Residents of rural regions may have higher and unique suicide risks. Newfoundland and Labrador (NL) is a Canadian province replete with rural regions. Despite an abundance of rural suicide research, heterogeneity in rural regions may preclude amalgamating findings to inform prevention efforts. Thus, exploring the unique needs of NL is needed. Importantly, health care providers (HCP) may afford unique perspectives on the suicide-related needs or concerns of rural life. We asked HCPs of residents of rural NL their perceived suicide risk factors, concerns, and needs for rural NL.

**Method:**

Twelve HCPs of rural residents of NL completed virtual semi-structured interviews. Interviews were analysed using reflexive thematic analysis [13,14].

**Results:**

HCPs noted individual, psychological, social, and practical factors linked to rural-suicide risk and subsequent needs. Findings highlight the unique challenges of residing and providing health care in rural NL and inform prevention and intervention efforts.

## Introduction

Suicide is a global health concern with over 700,000 deaths by suicide each year [1]. Canada is no exception, with approximately 12 Canadians dying by suicide each day [2] (Statistics Canada, 2020). Indeed, suicide is multifactorially influenced [3], underscoring the need for a multi-pronged approach to curb suicide risk. Research points to several unique contexts that may carry particular risk for suicidal ideations and behaviours (SIBs). Of note, individuals living in rural areas may be at a higher suicide risk compared to their nonrural counterparts [4].

Despite an abundance of research examining suicide in rural settings, a recent review of rural suicidology points to several key concerns in the field [5]. First, a lack of clarity and consistency in the definition of ‘rural’ limits the extent to which findings across studies can be integrated. Findings from this comprehensive review indicate that researchers often study heterogeneous populations but adopt the umbrella term ‘rural’ in reference to the population being studied. This may lead to conclusions and recommendations that do not fit within or across studies. Second, there are relatively few studies investigating differences in rural and nonrural regions with regard to suicidal ideations or non-death behaviours; instead, suicide death is primarily the focus in rural suicidology, despite ideations being more common and having the potential to be impairing across life domains [6]. For example, in Canada, approximately 0.01% of the population will die by suicide each year while 2.6% will have thoughts of suicide [2]. Accordingly, expanding the scope of rural suicidology beyond death is imperative to inform theory and augment prevention efforts. Last, that there is a dearth of studies investigating suicide in rural Canada, with a few notable exceptions [7,8]. Importantly, inconsistencies in rural definitions impede the ability to apply the existing literature to support rural Canadians. Furthermore, rural Canada is likely different from rural regions in other nations due to unique country-specific contexts (e.g., geographic, social, economic, and political climate). Along these lines, regions likely vary within Canada (e.g., across provinces) and within provinces.

Suicide rates vary substantially between Canadian provinces and seem to be particularly high for Newfoundland and Labrador (NL). Indeed, a recent time-trend analysis highlighted that the suicide rates in NL have increased three-fold from 1981 to 2018 [9], with current rates above 11 per 100,000 [2]. Importantly, over half of suicide deaths in NL are individuals from rural areas [8]. Understanding the unique suicide-related considerations relevant for rural NL is needed for prevention efforts.

One important way to gain insight is drawing from the perspectives of health care providers (HCPs). Indeed, HCPs play a key role in inquiring about suicidal thoughts [10] and are often the initial point of contact for mental health concerns. Thus, HCPs are integral to the identification and management of suicidal ideations or behaviours. As a result, HCPs can provide a ground-level and in-depth account of challenges with health service provision related to rural suicide. Some HCPs’ views related to suicidal ideations or behaviours have been examined in prior work [11,12]. However, this work did not specifically consider rural providers nor their perspectives of rural suicide risk. In line with the previous concerns regarding heterogeneity in rural definitions and regions [5], understanding the unique perspectives of HCPs in rural NL can inform how to best address the concerning suicide rates in the province.

The current study is, to our knowledge, the first to seek the perspectives of HCPs of rural NLs regarding factors that place rural residents at increased risk for suicide, which informs the potential unique needs of rural regions. Hence, the present study aimed to address the following question: What do HCPs perceive as suicide risk factors, concerns, and needs for residents of rural Newfoundland and Labrador?

## Method

### Participants

The current sample was derived from a larger online study examining perspectives, risks, and experiences with suicide among HCPs in NL. The larger study took place between July 22, 2021, and January 12, 2022. Of the 157 HCPs who completed the online set of questionnaires, 35 (22.30%) indicated that they would like to participate in a virtual one-to-one interview. Of these, 12 (34.29%) completed an interview. Interviews took place between May 20, 2022, and August 28, 2022. These participants were predominantly White (91.67%) and identified as women (91.67%). The average age of participants was 36.38 years (*SD* = 5.6; range = 28–43). One participant did not respond to demographic questions.

Of the 12 HCPs who took part in the study, six were social workers (50%), three were registered nurses (25%), two were psychologists (16.67%) and one was a paramedic (8.33%). The sample represented all four health authorities in NL at the time of the study: Labrador-Grenfell, Western, Central, and Eastern. Most participants resided in a rural region (*n* = 9; 75%) and primarily worked with individuals who likewise resided in rural regions. Specifically, the average proportion of patient rosters residing in rural regions was 97.25% (*SD* = 7.00); seven individuals indicated that 100% of their patient roster resided in rural regions. Importantly, given our research goals, participants self-determined what was considered ‘rural’.

### Measures

In addition to demographic questions, participants completed a semi-structured interview with the first author. Interviews lasted an average of 63 minutes (*SD* = 9.90 minutes; range = 36–92 minutes). Interview probes (see S1 Appendix) were relevant to the study’s goals; additionally, due to the semi-structured nature, interviews often focused on information that participants, by nature of their in-depth discussions, believed to be relevant to the study’s questions.

### Procedure

The study received ethics clearance from the University of Guelph’s research ethics board (REB#: 21-03-022) and the NL Health Research Ethics Authority (HREB#: 2021.077). In addition, consent to conduct research with HCPs employed by health authorities was obtained from all the provincial health authorities that existed at the time of the study.

As part of the larger questionnaire study, participants were recruited from advertisements posted to social media and emails sent to NL-based health listservs and HCP organizations. Interested participants could access the study by clicking a link within the advertisement. The participants in this study indicated their interest in a one-to-one interview at the end of the online questionnaire study.

Interested participants were emailed a consent form for review and a list of potential dates and times (90-minute blocks) to complete the interview. Participants emailed the first author to indicate their written consent and to book a meeting time. Participants were then sent a secure meeting link to a private and secure online WebEx meeting. During the meeting, the first author provided information about the study and participants were given opportunity to ask questions. Participants were then sent a link to a demographic questionnaire, which participants completed remotely via Qualtrics XM. All participants completed online interviews with recorded video and audio. Once the interviews were completed, participants were provided information on how to access the study’s results upon completion and given information about local mental health resources should participation in the study raise any personal issues or concerns. These resources were also sent in email format. The interview audio was transcribed by Otter.ai software and exported as text files. Files were checked for accuracy by the first author. The resultant transcripts (i.e., interview content) served as the data for analyses.

### Analysis

Interviews were analysed using reflexive thematic analysis (TA) [13,14]. The experiential approach to TA appropriately addressed the research question through predetermined steps to analysis [13] that seek to understand the essence of participants’ meaning and experiences relevant to rural suicide. Specifically, we followed the steps typical of a TA: 1) Becoming familiar with the text by reading through transcripts and re-watching the interviews to ensure accurate transcription and recording initial thoughts and ideas in margins of transcripts; 2) Begin to formally code smaller units of text into potential codes using NVivo software, which was an iterative process that involved collating data by potential codes; 3) Search for themes, which were determined by the frequency and saliency of participants’ language; 4) Themes were fine-tuned and finalised into a coherent thematic map; 5) Generate apt names and definitions that reflect the core meaning of each theme; 6) Last, write up the results of the analysis with text excerpts to illustrate the themes. The first author coded the interviews and the first and last authors met weekly to bi-weekly to discuss the analysis. All other team members contributed to the interpretation and final thematic map.

Importantly, the analyses were informed by a systems perspective (e.g., Developmental Systems Theory [DST]) [15]. Specifically, individuals are seen as being influenced by various components of a complex system of co-acting levels (i.e., bidirectional and reciprocal), which include biological, psychological, social, cultural, and temporal influences. All levels influence a person and, thus, warrant attention to inform suicide prevention and intervention.

### Data sharing

The recordings and transcripts analysed during the current study are not publicly available due to the sensitive nature and potential for identifying participants or their patients mentioned during interviews.

## Results

Several core themes and subthemes represent the interpretation of experiences of rural HCPs. What follows are explanations of these themes/subthemes, in addition to verbatim quotes from participants to demonstrate the essence and groundedness of the themes. The results are structured to start at the broadest level of influence and transition inward (i.e., from cultural → interpersonal → individual). However, given the nature of DST, themes often cut across levels of influence, marking the importance of reciprocal interactions among these levels [16].

**Fig 1:**
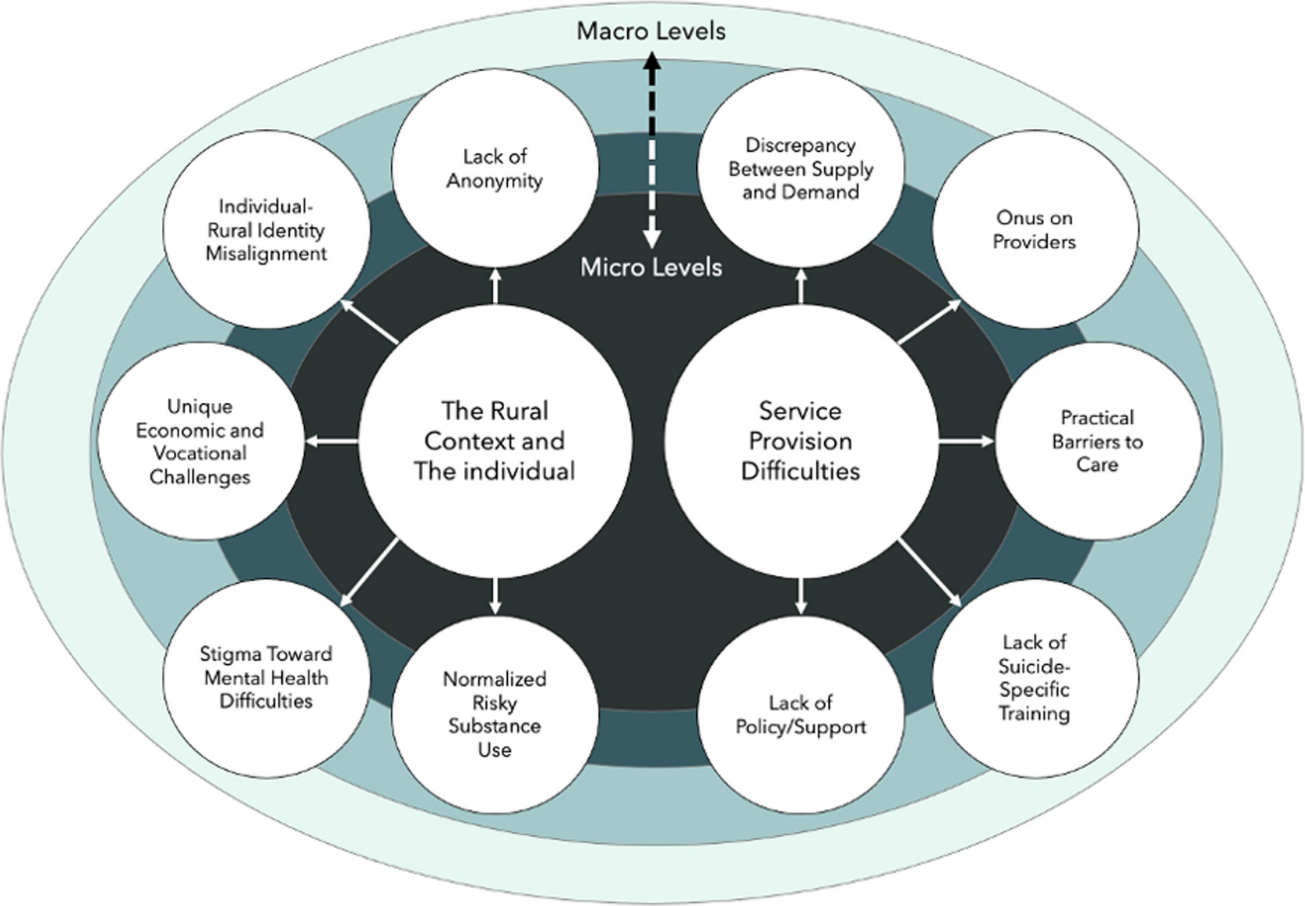
Thematic map related representing suicide risks and need.

Our research question focused on the risks, concerns, and needs related to suicide in rural areas. In line with this goal, participants were asked to speak to potential suicide risks in rural NL. Two important themes emerged from the interviews regarding suicide: i) The Rural Context and the Individual and ii) Service Provision Difficulties (see Fig 1). Each theme reflects the impact of various levels of a system that may impact the development of suicidal ideations or behaviours in residents of rural regions. Furthermore, themes may reflect the interplay between levels of influence (e.g., an individual with a health care system).

### The Rural context and the individual

The theme Rural Context and the Individual represents the broader sociocultural factors of rural regions that interact within individual-level factors that might increase an individual’s suicide risk. This theme was composed of five subthemes, which participants indicated were factors that increased suicide risk in rural NL, namely: a lack of anonymity, stigma toward mental health difficulties, normalized risky substance use, a misalignment between the self and rural identity, and economic/vocational challenges.

#### Lack of anonymity

Participants referred to a lack of anonymity resulting from small region dynamics that seemed to increase residents’ reluctance to engage with mental health services. For example, participants discussed the greater possibility for dual relationships with residents, which can impede help seeking. Indeed, participants mentioned that HCPs who also played other roles for rural residents (e.g., neighbour) could be a barrier to sharing mental health symptoms or distress, which may preclude help seeking and, thus, contribute to suicide risk. This was reflected in the following interview excerpt:

*…thinking about the clinics in rural areas, you know, maybe some people don’t want to go because the person working there is their neighbour, or, you know, is their sister-in-law, or whoever it is, you know. Maybe they don’t want someone close to them knowing their deep dark troubles. You know, there’s no anonymity.* (Participant 2)

Another individual similarly shared:

*I might be your doctor. But our kids might also be in hockey together. And I’m gonna see you in the supermarket, and I’m gonna see you at the gym, or I’m gonna see you when I’m out for a walk with the dogs. And we’re gonna say ‘hi’ at the post office, because we’re both members of this teeny, tiny community.* (Participant 1)

Others talked about the notion that community members are often aware of each other’s personal lives, as the following participant noted: *“But it can be really difficult because everybody knows your business all the time*. *And that is a really stressful thing for people”* (Participant 10). Having residents, including community HCPs, more involved or aware of the details of each other’s lives indicates how lack of anonymity may be an important obstacle in help seeking.

#### Stigma toward mental health difficulties

In line with a lack of anonymity, more stigma in rural areas compared to non-rural counterparts was also noted by HCPs as an impediment to help seeking for residents. Whereas a lack of anonymity focuses more on the dual relationships experienced in rural settings or the increased awareness between community members, this subtheme more pertains to the unique attitudes towards help seeking in rural regions. In line with this, one participant reflected on the views of mental health and suicide in rural areas, indicating that:

*I get it, mental health stigma is a universal issue right now. But it seems to be worse in rural Newfoundland. People just don’t seem to get just how mental health and suicide are real issues. And then they look down on other people who might, you know, need to get help for those issues. It’s very sad.* (Participant 3)

Another participant discussed ‘toxic masculinity’ and how it often interferes with an individual’s help seeking, and how rural NL seems to be particularly at risk: “*…the way we socialize men*. *It’s fucked up*. *Newfoundland and Labrador has a lot of*, *I hate to have to say this*, *but I’m gonna say it*, *toxic masculinity*. *Like culturally*, *Newfoundland and Labrador*, *we’re 20 years behind everybody else”* (Participant 4). This led to discussions on how to reduce suicide risk, which focused on reducing stigma and toxic masculinity:

*We should be talking about it at an early age and de-stigmatizing it* [mental health]*. And talking about the different types of supports that aren’t just therapy. Because if again, if I if my mind is correct, it’s mostly men killing themselves or dying by suicide. Oh, no, we don’t say that* [sarcastically]*. Then it’s about attitudes about therapy, support, talking about your feelings, the way men communicate. Those openings… I think that’s tangible, right, like, you know, talking about violence, talking about feelings. From a young age in the school system. The way we socialize men has to change.* (Participant 4)

Clearly, participants reflected on how stigma toward help seeking was an area of concern when considering suicide risk in rural areas.

#### Normalised risky substance use

Many participants discussed the normalisation of substance use in rural areas as playing a role in heightened suicide risk. Substances included alcohol, marijuana, and illicit drugs. For example, one participant discussed that alcohol is being used at a younger age by rural residents when compared to previous generations, *“I think everywhere rural has a huge alcohol issue*. *And it’s recognized and kind of accepted*. *But now the alcohol is in the younger crowd*. *And it’s… it’s scary to see the differences…see the changes over time”*. (Participant 6)

Others reflected on higher incidences of alcohol misuse, *“It’s like*, *all these small communities*, *you know*, *and I wouldn’t just say it’s in smaller communities*, *but there’s a lot of drug abuse and alcoholism”* (Participant 7). Another participant reflected on their suicide prevention efforts that focused on reducing alcohol consumption, *“So what we have focused on predominantly in our main safety pillar*, *is the correlation between alcohol consumption and these means of suicide*. *Because our research has also shown us that too… And that’s what we focused on was alcohol use*, *but again*, *there’s such a culture of*, *you know*, *drinking and drinking excessively*, *as opposed to social drinking*, *like you see in some other places”* (Participant 5).

Others mentioned drug use, such as one participant who discussed an increased suicide risk in rural areas because of drug use:

*And there’s so many different sides, every story, but a few of the kids are college-aged kids that left our site went home and committed suicide, there was drugs and alcohol involved. And obviously, they’re from rural* [removed] *and* [removed]*. …you’ll get people coming in that just needs to talk or vent and they’re like, ‘oh, a lot goes home and a lot of us drink and do drugs’. And that’s a big thing.”* (Participant 10)

One participant commented how seasonal workers, which many rural residents in NL are, may have extended periods without work, which is related to increased substance use. For example, when discussing the winter months, during which Newfoundlanders and Labradorians who work in the fish industry are not working: *“And then that tends to be when there’s more alcohol consumption*, *more drug use*, *and then more issues that would lead them to formal* [mental health] *service”* (Participant 11). Another noted about some rotational workers at risk for suicide by feeling isolated from family, and using alcohol to cope:

*Almost all the time that they’re home, they’re off time. And so they’re in the shed, and they’re partying, and they’re drinking, and they’re doing other things as opposed to being home, or doing things you would normally do with your family. So the family life is kind of turned upside down. And I think that does have something to do with their own anxiety, depression, maybe a feeling isolated, not connected… those kinds of things to their families*”. (Participant 5)

It seems, therefore, that participants consider risky drug use, particularly alcohol, as one factor that is related to later suicide risk.

#### Individual-rural identity misalignment

Participants also discussed how rural residents can sometimes perceive that rural culture does not align with their identity. For example, given there is less cultural variability within a rural area, a potential misalignment between someone’s identity and rural culture may be more likely than in non-rural areas and can be a factor in mental health difficulties and suicide risk. Rural culture was often described as marked by outdoor activities and that issues may arise if a person does not enjoy these activities. As one participant noted, “*not everyone is outdoorsy”* (Participant 2).

Another participant reflected on the potential struggles of growing up and not enjoying outdoor and related activities: *“Some kids don’t enjoy the typical rural activities like hunting*, *fishing… even if their parents are very invested in those activities*. *And you can imagine how hard it is for those kids*. *Like*, *there’s five kids in the community and they’re the odd one out*.*”* (Participant 3). Others spoke of the impact of having fewer individuals to connect with who may be like-minded; *“So if you’re from a rural area*, *you’ve got*, *like*, *this select amount of people that you can know*, *or like*, *you have a similar culture”* (Participant 8). This individual continued to explain that, *“If you’re an outlier in the culture*, *or the community… you don’t have a lot of options to feel supported”*. In sum, participants perceived the potential difficulty in connecting to others in the community and the broader rural culture as a potential risk for some rural residents.

#### Unique economic and vocational challenges

Participants often spoke of the changing economic and vocational landscape in NL. They commented on the faltering of typically relied-upon industries, such as the cod fishery, that has affected individuals’ identity and livelihood. For example, one participant noted that:

*A sense of purpose, a strong sense… healthy sense of identity, I think is really difficult in Newfoundland, because of how much it’s changed, and the lack of jobs and the way that the landscape of opportunity industry has changed so much over the last 30 years since the* [cod] *moratorium.* (Participant 4)

Another participant added how job loss interacted with other factors to increase suicide risk, *“And so things happen*, *you know*, *loss of jobs*, *and you’re coming home*. *And when you get drugs in the mix*, *and things like that it can be it can be hard when your entire mix there knows every everything about what’s happening”* (Participant 10). Others expressed that loss of employment impacts individuals’ sense of purpose and identity, *“There’s a lot of unemployment in rural areas and remote areas* …*and unemployment doesn’t necessarily inspire people to live”* (Participant 8). Similarly, another participant expressed:

*So you go through periods where there’s high rates of employment, a project comes in to the shipyard or something like that, and there’s, you know, a whole bunch of people employed and things are well, you know, it doesn’t seem like people’s mental wellness is affected as much because they’re financially stable. And then we go through very long periods of very high unemployment rates, and people out of work … So, we see a lot of men with like trades backgrounds, having to go away for work. And then, you know, that kind of a lifestyle has its own challenges, of course, but even for individuals who stay around and are employed, like in the local companies, when they get a layoff, I mean, their employment has been such a big, you know, indicator of who they are that when they’re laid off, they lose that sense of who am I as an individual because I’m no longer an employee.* (Participant 5)

As can be seen above, participants noted that the unique economic and vocational challenges of rural NL lead to numerous negative outcomes, all of which may increase the risk of suicide.

### Service provision difficulties

Similar to the first theme, the second also comprised subthemes that focused on difficulties associated with providing care to rural residents. Specifically, participants pointed to a discrepancy between supply and demand for suicide-related mental health services, practical barriers to care, a lack of suicide-specific intervention training, a lack of policy/support from managers, and the onus on the provider for training.

#### Discrepancy between supply and demand

Some participants reflected on the lack of professionals available to serve the mental health needs of rural residents. Participants discussed a lack of physicians, psychologists, nurses, and other professionals who play a role in suicide prevention and intervention. For example, one participant noted:

*Take it from the top doctors on down, there’s a lack of doctors, when you’re waiting seven months for referral. And that’s going on here. They get referred to a counsellor and it’s a seven month wait to see him… or you see a suicide risk come in. They’re referred to a counsellor, but sorry, the counsellor is unavailable for two weeks. I’m like, this can’t wait two weeks, and you send them home with a parent that may or may not have to work or whatever… there’s a lack of actual warm bodies.* (Participant 6)

Others talked about a lack of mental health professionals in their region such as psychologists. Participants often mentioned a lack of public mental health services, which limit who can access the already limited number of psychologists in the province.

*We need the government, not just the Newfoundland government, but the federal government to fund psychologists… if I had to avail of a psychologist, I’m okay. I got medical and dental insurance. So my insurance will pay up to maybe, I don’t know, seven or eight hundred dollars, you know, you might get four or five sessions. Most people don’t have that.* (Participant 7)

The desire to have more appropriately trained professionals serving within or near rural communities was also shared by participants. For example, one participant stated, *“They need the professionals there in the communities*. *So I think they need access to these professionals*, *mental health professionals*, *there within*, *you know*, *an hour drive*, *maybe”* (Participant 2).

Others discussed the reduced appeal of working in rural mental health care, which contributes to a mismatch in supply and demand. This was illustrated by the following individual: *“But if those positions aren’t filled*, *because nobody wants to work in rural health care*, *and in some communities*, *it just… the service doesn’t exist”* (Participant 1).

From the perspectives of the participants, there is a lack of services available to residents of rural NL. Participants noted this was the case for all health care professionals. This lack of health services may be due to lower service allotment and the difficulty in recruitment and retention of health care professionals in rural regions.

#### Practical barriers to care

Participants also discussed the barriers experienced by HCPs in providing, and rural residents accessing, mental health and suicide-related services. For example, participants indicated that rural Newfoundland and Labrador has barriers to service provision, such as reduced technology and connectivity, poor weather conditions, and lengthy drives to provide or receive services. As one participant noted, *“Even just having to drive two hours to a home visit in the middle of March*. *Like you don’t have cell phone coverage*. *The weather’s really bad*. *There’s a lot of wear and tear in your car”* (Participant 1). This individual continued to explain that transportation is an ongoing barrier, *“Yeah*, *there can be a Doorways* [mental health walk-in service] *clinician in your community*. *There can be a walk-in clinic*, *there can be a physician*. *But if you can’t get to them*, *you don’t get service*.*”*

Several participants referred to the technological challenges associated with mental health and suicide-related care in rural NL. In line with this, they noted that, while beneficial for some, telehealth was not a feasible method of receiving services for a large proportion of rural Newfoundlanders and Labradorians. For example, one individual noted:

*And we’re really limited in what we can do to, to reach out because we’ve got all this wonderful technology that COVID brought us, you know, all the Facetimes and meetings like this, which is amazing… but it’s absolutely useless if you don’t have an internet connection, or a phone or a laptop… which a lot of our clients don’t have access to.* (Participant 10)

Others reflected on their time living in rural areas to describe technology barriers, *“Yes*, *I’ve lived in a couple of different areas where the internet is not super*. *And this was probably 10 years ago*. *So I don’t know… things have changed*. *But I’ve worked in areas that you know*, *there’s no cell reception”* (Participant 2). Another individual noted, *“I lived in one area that had dial-up internet*. *And this was in… 2013*, *so not long ago*. *I couldn’t load the news*, *let alone sign onto a video chat*. *So how could you expect a patient to do it*, *especially if they’re in crisis*?*”* (Participant 3).

Another participant noted that technological barriers are quite pervasive and in potentially unexpected areas, such as close to larger urban regions in NL, *“I mean*, *we have patients*, *even in the St*. *John’s region*, *they don’t have phones”* (Participant 1). It appears that numerous barriers exist that impede the availability and provision of health services to rural Newfoundlanders and Labradorians.

#### Lack of suicide-specific training

The third aspect of service provision difficulties involved a lack of background, training, and opportunities for suicide-specific care. Participants often noted the limited training received through their formal education or by their current employers. Specifically, while some felt comfortable identifying suicide warning signs, there was little training regarding follow-up to suicide risk. As a result, individuals were often referred to emergency services, including hospitalization, which could create additional challenges in consistent, long-term suicide-related care. As clearly noted by one participant, *“So there’s no training*. *We need training*. *People aren’t even talking about it”* (Participant 12).

Individuals spoke about the lack of time or incentive to seek formal training: *“There is no incentive to go and do training*, *specialised training ‘cause you have to pay for it yourself”* (Participant 4). This same individual spoke about how underqualified they are at their position, yet were expected to provide evidence-based treatment without training:

*People should not be doing this job with a Bachelor of Social Work, put that in your paper. I should not be doing this job. The only reason I’m good at my job is because of my personality and the amount of reading I’ve done in my life, my own personal experience… I still don’t think I should be doing the job.* (Participant 4)

The fact that there was no mandatory training for suicide intervention was common across participants. Another participant noted *“…it’s unreal*, *that I can’t believe that* [suicide intervention training] *hasn’t become a mandatory”* (Participant 6). Participants also spoke to the larger issue that the professionals with extensive training are simply not available to rural regions, *“A big problem with it is that the people who I think really are trained are just not available*, *always in rural areas”* (Participant 2).

Some participants indicated that they completed Applied Suicide Intervention Skills Training (ASIST). However, ASIST is a short-term crisis identification intervention that seeks to then connect the individual in crisis to longer-term care, which is a critical component of suicide management. For example, one participant noted that ASIST is a community approach and by providing this as the main intervention received by HCPs, they are no different than general community members:

*…the only training that we’ve really had, or that we’re offered is ASIST. …which I did when I was a student in my undergrad, and maybe at the time, it was a fitting type of training for the place that I was at. And for where I was in terms of like being a student, and also a community member, just having an awareness of like reacting suicide and things like that. But it’s still considered by* [health authority] *as like the training for professionals mental health clinic, which I would disagree with. You know, it’s not really preparatory, or comprehensive enough, as somebody who should know more than a community member… if it’s being offered to anybody who wants to have it, you then it’s saying like, you know, as much as anybody who is in the community. Right? Which we’re supposed to be specialists, more so than somebody just off the street, right?* (Participant 8)

Ultimately, participants expressed a lack of suitable and specialized care related to suicide. Despite this, participants noted their willingness and desire to complete training, which was sometimes impeded by little incentive or support.

#### Lack of policy/support

Participants often described that there was insufficient or conflicting policy regarding suicide prevention. For example, one participant reflected on their time working in various health authorities in NL. At the first authority, there was substantial policy and guidance around suicide assessment. However, when they moved to another health authority, they perceived that there was inadequate and desultory guidance for working with at-risk individuals. While discussing the current practice in their workplace, they noted, *“Like*, *how do we react*? *Because I’ve worked here for four years*, *where’s my package of assessments*? *It’s literally*, *I’ve worked here for four years*, *someone* [manager] *told you literally say to the person* [patient], *‘do you have a plan*?*’”* (Participant 4). Another individual spoke about wanting a more comprehensive assessment strategy but being denied financing to purchase these assessments: *“They* [manager] *will say like*, *‘oh*, *well*, *you don’t need to do this test… you can tell if they’re okay or not*, *or base all just kind of on subjective information’”* (Participant 8). Another participant expressed similar concerns over a lack of formal suicide assessment policy:

*Coming here, I was shocked and all that there is no formal paperwork for that kind of stuff. There’s not even a rating, one that we fill out on a policy level. So I was kind of shocked by that. Because I think there is quite a high rate of new suicidal… suicide or suicide attempts in Newfoundland and Labrador.* (Participant 3)

Another discussed their surprise when they learned that there was a lack of suicide-related policy in one specific health authority. For example:

*When I came to* [health authority] *and to work for* [location]*. I was like, oh, so what’s the suicide policy? Because the very first day I worked here, I had a suicidal teenager. So they were like, oh, no, they’re not really is one* [policy] *you just like, you know, ask them if they have a plan to kill themselves. And like if you think they need to go to the hospital, tell them to go to the hospital. And I was like, don’t we? So like, I think* [health authority] *is underprepared, or under-policied, or under-structured when it comes to suicide.* (Participant 4)

Others reflected on how management may not understand the unique challenges of rural practice because they themselves are not located in rural regions. As a result, they may not be able to relate to HCPs’ experiences or provide relevant support:

*I mean, your manager might be in another part of the region, but you never physically see them ever. And you might have to wait a week to get a call back to consult on something. And if you’re sitting in somebody’s house, and they are saying, ‘I don’t want to live anymore’, you have a week. And I mean, that’s certainly not every manager, but there are some that are just inaccessible… and why are they inaccessible?* (Participant 1)

Another HCP discussed how smaller mental health teams in NL are typically comprised of and managed by social workers, who may have a different approach to suicide assessment and intervention when compared to other health care professionals. This participant, a non-social worker, indicated that this can create challenges when seeking support, consultation, or guidance from managers. For example, a participant expressed, *“my manager is a social worker who isn’t trained like me… they don’t approach assessments or treatment like I would*. *I hate to say it*, *but sometimes it feels like what’s the point of consultation when we view the major issues as different”* (Participant 3).

Another participant noted the lack of consultation structure or integration between units that hinders supporting rural individuals. For example, this participant recounted being in a counselling session with a patient who indicated that they were planning suicide. After some discussion, the patient quickly and unexpectedly exited the telephone session. The participant reflected that, *“And so for me*, *because I am not part of the mental health team*, *I was like*, *frig*, *like*, *what do I do*? *So luckily*, *I walked down the hall*. *And it’s like*, *‘Hi*, *I need to consult*, *I need to consult’*. *And that should really be a formalized structure*, *instead of me hoping that somebody is at their desk”* (Participant 1).

Indeed, as illustrated above, participants highlighted several difficulties that can arise from less cohesive relationships with managers and a lack of structure or policy regarding suicide prevention.

#### Onus on provider

The last aspect of service provision difficulties focused on the provider bearing responsibility to acquire suitable suicide intervention training. Participants commented on the lack of formal and required trainings on suicide assessment and interventions. For example, one individual remarked that *“We had one small… we call them learn modules*, *which is like a*, *you know*, *we go into our learning interface*. *And we do… it’s like probably a 20-minute video on suicide*. *And that’s not compulsory*. *That is if you choose to do it”* (Participant 7). Others discussed how learning and professional development are not feasible in the rural public sector because of an increased workload:

*Like, unless you’ve built the skill yourself, unless you’ve done the research, which from a public services kind of perspective, we don’t have the time to do it*. *Like there is no time built into my day or week to research things… I have a waitlist, there are people who are desperate for service. So it’s like, do I take two hours to… to read something? Or do I see two individuals who are desperate for service?* *(Participant 1)*

Similarly, one participant explained that even if training were available, *“…it wasn’t compulsory*. *And you know*, *for someone working Monday to Friday*, *eight to four*, *you can’t commit to that stuff*, *right*? *Because they aren’t going to give you the time to do it*” (Participant 3). Ultimately, some participants perceive that getting suitable suicide-related training conflicts with the numerous other demands of their work and, inevitably, is given less priority over these other demands.

## Discussion

Suicidal ideations and behaviours may disproportionally and distinctively affect rural residents [5], highlighting the importance of tailored prevention and intervention efforts. The present study sought to understand the perspectives of healthcare providers (HCPs) for rural residents regarding suicide risk factors in rural regions. The unique suicide risk factors for Newfoundlanders and Labradorians, as identified by HCPs taking part in this study, have implications for intervention efforts to, ultimately, reduce suicides.

The participants’ perceived suicide-related risks seemed to be organised and may be best reflected through a systems approach, wherein there are macro- (e.g., rural culture) and micro-level (e.g., HCP and rural resident factors) considerations as well as interplay between biological, psychological, and social factors [17,18]. Indeed, themes seem to cut across and represent the interactions among levels of influence.

First, there are several *Rural Context and Individual* factors that HCPs highlighted as engendering suicide risk for rural NL residents. Rural communities were described as sometimes lacking anonymity. Past research highlights a nuanced relationship between anonymity, stigma, and disclosure. For example, individuals with higher degrees of embarrassment about their illness were more likely to disclose in an online and anonymous setting, marking the utility of anonymity for some individuals [19]. Others have noted that the type of mental disorder and severity of symptoms may impact an individual’s likelihood of disclosing in anonymous versus identifiable outlets [20]. Regardless, HCPs in this study perceived that a lack of anonymity is closely linked to stigma and may be a deterrent for seeking formal mental health and suicide-related services. Indeed, there is evidence from both service recipients and providers that stigma for mental illness or mental health difficulties is associated with reduced help-seeking, particularly for rural areas [21,22]. Although telehealth may mitigate this barrier by allowing individuals to have appointments from the privacy of their own home, the technological barriers of some rural residents frequently make telehealth a frustration versus a benefit. For example, one HCP in this study recounted having appointment cards thrown back in their face by a patient who expressed the uselessness of a video appointment because the patient did not have cellular or internet access.

In addition to lacking anonymity, HCPs highlighted that substance use, and in particular alcohol, is a common and normalised element of rural culture. Substance use has previously been linked to rural living [23]. Importantly, a recent meta-analysis of over 2 million individuals within 30 studies indicated a positive relation between alcohol use and suicide attempt and death [24]. Given that alcohol abstinence may not a be a feasible long-term strategy for many individuals, particularly if it is ingrained in a given culture, a harm reduction approach tailored to rural residents may be more beneficial for improving physical and psychological functioning, including reducing suicide risk [25,26].

HCPs also discussed factors related to individual residents of rural areas that may increase suicide risk in these areas. Here, they discussed a discrepancy between an individual’s identity and the larger rural culture as a potential suicide risk factor. While non-rural areas may have more cultural diversity with which residents can connect, rural areas present fewer opportunities for individuals who do not align or match with the dominant culture of a region. Indeed, this mismatch between individual beliefs, values, and preferred activities and those of the dominant culture, (i.e., cultural consonance) has been linked to psychological distress [27]. Furthermore, a disconnect between the individual and their community may be interpreted as one facet of the broad construct *thwarted belongingness* (e.g., loneliness and a lack of meaningful reciprocal relationships), which is purported to be an important contributor to suicidal ideation [28,29]. Similarly, other ideation-to-action suicide frameworks highlight the importance of perceptions of connection to people, but also more broadly to roles, work, or life purpose [30].

Beyond individuals’ cultural dissonance, HCPs reflected on the unique economic and vocational challenges of rural Newfoundlanders and Labradorians as risks for suicide. Specifically, they reflected on reductions in industry, such as the cod fishery; “Newfoundland has always been associated with fish, which dominated the economy for hundreds of years” [31, p. 411]. The relation between unemployment and suicide has been noted internationally [32], with estimates indicating that for every 1% increase in unemployment, suicide rates increase by about 1–2% [33,34]. Therefore, for perceived suicide risks articulated by study participants, policy promoting financial and workplace stability for rural residents of NL may serve as suicide-mitigating factors.

Additionally, HPCs in this study reflected on rotation work as a vocational factor that may contribute to rural suicide risk, pointing to rotational workers often spending significant time away from friends and family, which may increase isolation. Isolation could result in lower perceptions of belongingness and connectedness, which are believed to be important causes of suicide [29]. Indeed, when NL industries decline (e.g., fishery), individuals may be required to work outside of their community or, at times, province. Extended periods away from family and friends, often on work camps or sites, may engender social disconnect from important support systems. HCPs noted that rotation work interferes with important social spheres, which may increase suicide risk. This aligns with contemporary suicide theory in which increased disconnect or decreased belonginess may be contributors to thoughts of suicide [29,30].

Several *Service Provision* factors are also relevant to understanding and addressing suicide risk in NL. HCPs in this study noted a *Discrepancy Between Supply and Demand* of suicide prevention and intervention resources in their regions. HCPs pointed to vacant positions of HCPs in rural areas, despite a steady demand for services. As a result, many residents are faced with extended wait times or lengthy travel to access services. Some residents simply cannot access recommended care, highlighting the inequalities in suicide-related service provision for rural residents. The perspectives of HCPs align with recent data that notes that rural Canadians comprise 17.8% of the total population [35], but 12.8% of family medicine physicians and 2.2% of specialist physicians serve rural regions [36]. Furthermore, the Eastern Health region of Newfoundland, which contains what might be considered the province’s only urban center had approximately 162 physicians per 100,000 individuals in 2021, marking a 1.1% decrease from 2017. However, Labrador-Grenfell, Western, and Central regions had 72, 99, and 73 physicians per 100,000 in 2021, marking decreases by 18.9%, 16.5%, and 2.6% since 2017, respectively [36]. The impact of the recent amalgamation of NLs four health authorities on service inequality remains to be seen.

Related to supply-demand discrepancies, another health care systems factor mentioned by participants pertained to *Practical Barriers to Care* that impede suicide-related care in rural NL. In particular, HCPs discussed the travel required to access healthcare, which is not feasible for all individuals. Others noted technological barriers that make telehealth or other electronic services frustrating, if not futile. Although there are noted benefits to mental health care in the digital age, such as self-initiated psychoeducation, fostering social connections, and reduced stigma in accessing services [37], rural residents in some areas of NL may not be afforded these potential benefits due to inadequate internet or cellular services.

HCPs also drew attention to a general *Lack of Suicide-specific Intervention Training* as a service provision barrier. A lack of training may be linked to more suicide risk-related hospitalizations. This is concerning given the potential for iatrogenic effects linked with hospitalization for suicidal ideations or behaviours. For example, hospitalization is linked to coercion and a loss of autonomy, all which may be linked to increase suicide risk or traumas [38]. Furthermore, hospitalization may not reduce long-term suicide risk and, in some cases, increases risk [39]. Thus, hospitalization is a last-line measure for at-risk individuals in some evidence-based treatments (i.e., avoiding hospitalisation in the Collaborative Assessment and Management of Suicidality [CAMS]) [40]. Increased hospitalizations may be linked to inadequate training in the assessment and management of suicide on the part of clinicians. Indeed, while some HCPs noted that they received some training, such as ASIST, the education on and implementation of evidence-based suicide interventions were said to be largely lacking in rural NL. In line with the subtheme that highlighted a disconnect between supply and demand, professionals with specific training in suicide-related interventions or opportunities to obtain evidence-based training seem needed in rural NL. Professionals, such as registered psychologists with comprehensive practitioner training that includes suicide risk assessment and management, may be in prime positions to fill the void with evidence-based treatments for suicidal ideations (e.g., CAMS) [41] or behaviours (e.g., Dialectical Behavior Therapy [DBT]) [42]. Unfortunately, difficulties in hiring such professionals may be a barrier to providing suitable services to residents of rural NL.

Additional service provision difficulties expressed by HCPs involved lack of consistency and limited support from policy and managers. Insufficient managerial support has been identified as a risk for burnout, including emotional exhaustion, depersonalization, and lack of personal accomplishment [43]; HCPs are no exception to the negative association between supervisor support and burnout [44]. Importantly, this may impact suicide-relevant care as, for example, HCPs may miss nuances in a patient’s presentation that would be considered a flag for suicide risk. Furthermore, some HCPs noted that managers and/or supervisors work from separate, distant, and, sometimes, non-rural regions, making them less exposed to and aware of the unique challenges associated with rural practice. This difference in physical location also makes them less available for immediate consultation. Thus, increasing the level of support or face-to-face interactions between rural HCPs and their supervisors or managers may help improve HCP well-being and reduce burnout, which may in turn improve service provision to rural residents.

The last subtheme relevant for service provision reflected HCPs perception that, given little direction from policy and/or managers, the onus is on them to seek out and complete suicide-specific training. Given rural HCPs high caseload, having to seek out additional training may be quite difficult. This may be complicated by the lack of psychologists providing outpatient public health services in NL, which was expressed by many of the participants. Psychologists’ ethical practice guidelines require them to maintain an up-to-date breadth of knowledge relevant to their practice, including suicide interventions [45]. As such, they may be afforded time to pursue additional training related to suicide as a component of their positions in health care.

### Implications

**Research.** The use of a systems approach to understanding suicide risk may carry utility in future research. HCPs in the present study highlighted numerous factors that represent interacting levels of rural residents’ systems including cultural, social, and psychological (e.g., how individuals fit within the majority culture in their region). While the probes that were prepared for interviews inquired about participants’ perspectives on suicide-related risks at various levels of a system (e.g., individual, culture), participants seemed to naturally discuss aspects of a system with connected and transactional levels. This aligns with contemporary views of suicidal thoughts and behaviours, which are complex and influenced by multiple reciprocally interacting factors [46]. Researchers ought to continue exploring various levels of rural residents’ system to better understand indirect, direct, and causal risks of suicidal thoughts and behaviours. Importantly, creating models that incorporate the interactions of multiple levels may best describe, explain, and predict suicide. For example, developmental systems approaches [15] may provide a framework to comprehensively study suicide risk in rural regions. Conversely, focusing on individual or subsets of risk factors may do little for long-term suicide prevention efforts (see [3]). Incorporating rural-specific suicide risks into suicide theory or explaining how these factors manifest in theory-relevant constructs may strengthen the explanatory power of the theories.

**Policy.** Policy makers and influencers can draw on the results of this study to implement ground-level changes for HCPs. Indeed, barriers to care, lack of policy or support, and personal HCP responsibility for training were all discussed by the participants during the interviews. Other HCPs noted a lack of access to mental health professionals in rural regions, namely psychologists and psychiatrists, that causes a discrepancy in supply and demand. Additional recruitment initiatives and incentives to attract professionals to health care positions involving rural care is needed. A potential avenue for future research is to investigate HCPs perspectives on the factors linked to accepting or leaving a position in rural health care, which may help inform policy changes needed to recruit and retain HCPs. Furthermore, ensuring opportunities and time for current rural HCPs to complete professional development and related training would be beneficial. These factors are amenable at a policy or health authority level. Finally, HCPs highlighted the disparities in digital and virtual health provision in NL (e.g., barriers accessing technologies needed for virtual services). As a result, policy makers and influencers should work towards health services equity for those in rural and remote areas. For example, relevant policy can ensure that rural residents have access to digital and virtual health services in their homes or through central services hubs (e.g., leveraging health clinics or schools that have more reliable technology).

**Clinical.** HCPs highlighted several factors they thought to be important risk factors for suicide and other clinical professionals could benefit from awareness of the various cultural, social, and psychological factors in suicide risk assessment and, potentially, intervention. For example, empirically-informed approaches to reduce the consumption or the negative impacts of alcohol and other substances may benefit rural residents by reducing the likelihood of suicide attempts after acute alcohol use [47] or learning alternative coping skills. Motivational interviewing [48] may be a suitable intervention for substance use by helping individuals work through potential ambivalences about substance use. Otherwise, harm reduction approaches may be beneficial [25].

While some individuals present with thoughts, emotions, or behaviour indirectly linked to suicide, others may present specifically with direct suicidal thoughts or behaviours. While recognizing the challenges associated with rural health care, which is informed by the results of this study, it is recommended that clinicians implement evidence-based treatments for suicidality including DBT [42], CAMS [41], and Cognitive Behavioral Therapy for Suicide Prevention [49]. This largely begins at a policy and management level; HCP opportunities for training (i.e., allotted time and funding) must be given priority by administrators and policy makers. Practicing in these modalities will afford HCPs of rural residents the opportunity to better serve their patients and themselves. For example, perceptions of adequate suicide-related training are positively associated with self-efficacy and negatively associated with anxiety about working with patients [50]; however, these relationships are cross-sectional and do not indicate a causal effect.

In addition, reducing hospitalization as a first-line response to suicide risk may prevent a host of iatrogenic outcomes [38], some of which were discussed by HCPs in this study. Current approaches to managing suicide focus on reducing hospitalization (e.g., CAMS) [40] and increased collaboration between service settings (e.g., community health, emergency departments) may reduce the use of unnecessary hospitalisation.

**Limitations.** The results of this study must be interpreted within the context of its potential limitations. First, the impact of the COVID-19 pandemic on health care professionals cannot be underestimated. HCPs internationally have experienced increased burnout [51] and depressive symptoms [52], and an overall negative impact on well-being [53]. This may impact the willingness and ability of HCPs to participate in research; our study is no exception. Indeed, one participant was called to the emergency room during the interview, highlighting the competing and ongoing demands on HCPs. Furthermore, the rate of attrition in this study (34.29% of those indicating interest in an interview completed the interview) may reflect the high degree of fatigue and competing demands for HCPs in the current COVID-19 context. Within this context, the perspectives of HCP of rural residents may not be completely captured by this sample.

Second, sample characteristics may have limited the diversity of our results. Specifically, our sample was composed of predominantly White individuals who identified as women, whose perspectives likely vary from minority populations in NL. Furthermore, the sample contained no primary care physicians, who also likely receive suicide-related disclosures when working with rural NL residents. For example, one study of suicide disclosure indicated that 17% of the sample was asked about suicide by a medical doctor, whereas 4.2% were asked by a nurse [54]. Having additional professions or specializations in the sample to capture the range of services available–or unavailable–to people in rural NL may have identified perspectives not fully represented in this study.

Third, not all individuals disclose suicidal thoughts or behaviours to another. Indeed, one study investigating disclosure in a clinical sample reported varying reasons for (e.g., receiving help or support) and against disclosure (e.g., fear of rejection or hospitalisation) [55]. As a result, the HCPs’ experiences as captured by this study may only reflect perceived suicide risks for those who have already disclosed suicide to the HCPs. A logical extension of this study is to draw on the perspectives of rural residents with lived experience of suicidal thoughts or behaviours. This may partially bypass the potential limitation related to disclosure in this study and expand to our understanding of rural suicidology.

### Concluding remarks

Suicide is a global health concern impacting thousands of Canadians each year [2]. Research has highlighted the increased risks faced by residents of rural areas [4]. However, rural has largely been a heterogeneous variable within the suicidology literature, with little consideration of the social aspects of the construct [5]. Newfoundland and Labrador is replete with regions that are typically considered rural and may face unique cultural, social, and economic risks for suicide. Given fewer mental health specific services, suicidal thoughts or behaviours may be disclosed to both general health care providers, such as nurses, or specialized providers, such as psychologists or social workers. Given this, HCPs are in a prime position to speak to the unique suicide risk factors for residents. This study provided an in-depth exploration of the experiences of HCPs for residents of rural NL regarding the perceived suicide risk factors for residents of rural regions.

Our study has several key findings that can benefit research, policy, and clinical practice. Several suicide risk factors were described by HCPs, ranging from macro- (e.g., broad rural culture) to micro-level (e.g., interactions between HCP and patient). The factors highlighted by HCPs in this study may provide clinicians with assessment and intervention focal points. Additionally, increased support, resources, and guidance from management and policymakers may facilitate HCPs’ ability to provide effective care for their patients and, thus, reduce their risk of suicide.

## Supporting information

S1 Appendix Focus group probing questions. (DOCX)
