# Supplementary material for: Rural suicide in Newfoundland and Labrador: A qualitative exploration of health care providers’ perspectives
Source: PLoS One. 2024 Aug 12;19(8):e0306929. doi: 10.1371/journal.pone.0306929 (PMC11318929; doi:10.1371/journal.pone.0306929)
Supplement: S1 Appendix — (DOCX) [file pone.0306929.s001.docx]

Supplementary Document 1

Focus Group Probing Questions

Q1: How would you define a rural region? What are the unique things that make a region rural?

Q2: How is a suicide an issue in the rural regions you serve? What are the economic, policy, geographic, and rural resident/community-level factors that need to be considered and why?

Q3: Broadly, what types of suicide, assessment, or treatment training do healthcare providers receive? What type as training is neglected or needed? How would these support healthcare providers supporting rural residents experience suicidal thoughts or engaging in suicidal behaviors?

Q4*:* What are the barriers, challenges, or deficits in supporting rural residents that contribute to suicide risk? For example, at social, economic, cultural, and structural levels.

Q5: What are the strengths of rural residents and communities that help mitigate suicide risk?

Q6: What are the major strengths of rural health care providers that help mitigate suicide risk?

Q7: What do rural residents need to mitigate suicide risk? For example, at social, economic, cultural, and structural levels.

Q8: What do rural health care providers need to mitigate suicide risk? For example, at social, economic, cultural, and structural levels.
